# The universe of ANA testing: a case for point-of-care ANA testing

**DOI:** 10.1007/s13317-017-0093-6

**Published:** 2017-03-21

**Authors:** Konstantin N. Konstantinov, Robert L. Rubin

**Affiliations:** 1 Division of Rheumatology/Department of Internal Medicine, University of New Mexico Health Sciences Center, 1 University of New Mexico, Mail Stop MSC10-5550, Albuquerque, NM 87131 USA; 2 Rheumatology Section, Raymond G. Murphy VA Medical Center, 1501 San Pedro SE, Albuquerque, NM 87108 USA; 3 Department of Molecular Genetics and Microbiology, University of New Mexico Health Sciences Center, 1 University of New Mexico, Albuquerque, NM 87131 USA

**Keywords:** Antinuclear autoantibodies (ANA), Point-of-care (POC) testing, Electrochemical biosensor

## Abstract

Testing for total antinuclear antibodies (ANA) is a critical tool for diagnosis and management of autoimmune diseases at both the primary care and subspecialty settings. Repurposing of ANA from a test for lupus to a test for any autoimmune condition has driven the increase in ANA requests. Changes in ANA referral patterns include early or subclinical autoimmune disease detection in patients with low pre-test probability and use of negative ANA results to rule out underlying autoimmune disease. A positive result can lead to further diagnostic considerations. Currently, ANA tests are performed in centralized laboratories; an alternative would be ANA testing at the clinical point-of-care (POC). By virtue of its near real-time data collection capability, low cost, and ease of use, we believe the POC ANA has the potential to enable a new paradigm shift in autoimmune serology testing.

## Significance of ANA testing

Autoantibodies are essential serological markers that define and classify most autoimmune diseases. Testing for total antinuclear antibodies (ANA of undefined specificity and which includes anti-cytoplasmic autoantibodies) has become an invaluable tool at both the primary care and subspecialty settings as a window into further clinical investigation. The presence of total ANA triggers follow-up diagnostic studies for specific autoimmune disorders that are part of the diverse clinical landscape seen in rheumatology, neurology, oncology, and in infectious, pulmonary, and renal diseases among others. In many of these conditions ANA constitutes part of classification and diagnostic criteria for those diseases. The total and ANA sub-serology tests also facilitate differential diagnosis and its refinement [1], predict incipient disease [2], indicate disease severity or impending flares [3], serve as prognostic markers for further organ involvement [4], monitor efficacy of therapy [5], and asses induction of autoimmunity by drugs [6].

The present article attempts to summarize current trends in ANA serology testing by diverse physician groups and to provide ideas on how point-of-care delivery of ANA results may produce the framework, knowledge, and practices which may benefit patients, providers, and the health care industry as a whole. While methods for ANA detection have markedly evolved in recent years, newer methodologies generally require expensive instrumentation and/or central clinical laboratories. An inexpensive and reliable point-of-care device for ANA testing could be applicable not only in communities with modern health care infrastructures but also in more resource-poor settings that struggle with provision of medical care and high burden of disease.

## Changes in ANA ordering pattern

There are many factors, which determine a physician’s test ordering practices. These include diagnostic, prognostic, and therapeutic factors, as well as patient-related factors (reassurance), doctor-related factors (individual clinical experience and confidence in clinical judgment; fear of litigation), and organization-related factors (test availability; institutional policies) among others [7]. Regarding ANA testing, the current increase in ANA requests may also be a consequence of two additional factors: (1) repurposing of ANA from a test for lupus to a test for diverse autoimmune diseases and (2) the expanded and central role of the primary care physician (PCP) in the health care delivery system. Today, ANA test ordering practice follows one of the following patterns:

1. Intent to diagnose (subspecialists)

ANA testing is routinely performed during initial evaluation of patients with increased pre-test probability for autoimmune disease [8], the consequence of which includes substantial morbidity, mortality, and general health care costs. Negative ANA can provide a quick way for ruling out disease, while positive ANA can lead to further diagnostic consideration and sub-serology testing.

2. Intent to refer (primary care physicians, PCP)

According to the CDC and Prevention’s National Ambulatory Medical Care Survey [9], more than half of doctor’s visits are made to primary care offices in outpatient settings. A PCP can have long-standing relationships with their patients, and it is only natural that they are at the center of the referral decision process. Important triggers for a referral recommendation by a PCP to a specialist are the clinical characteristics associated with the patient’s presenting health problem [10]. In the case of suspected autoimmune disease, these may be systemic but vague and non-specific complaints, and tests like ANA capture information that are needed to justify referring the patient to a specialist. Negative ANA can provide a quick way for ruling out disease.

3. Intent to case find for early disease prevention (PCPs)

In an insightful review and commentary on ANA testing [11] M. Fritzler points out that the early detection of autoimmune disease is critical to ensure that treatment is promptly administered to minimize the development of disabling conditions. Case finding should be proactive; it usually works in low pre-test disease probability situations, and uses symptoms, risk factors, and/or demographics at an individual level to inform assessment, management, referral, and education [12]. This approach is promising for diseases with long preclinical periods (a feature of most autoimmune disorders). It attempts to achieve pathology prevention by avoiding factors that trigger disease or using therapy that modules the destructive process before the onset of clinical symptoms [13, 14]. The existing literature on the predictive significance of ANA-associated rheumatic diseases justifies active case finding in practice as one of the main goals of the PCP when ordering an ANA test [11].

4. Intent to reassure patient (PCPs and subspecialists)

Testing with the intent of “reassurance” is not infrequent, despite the fact that when done for symptoms with low risk of serious illness, it is doing little to decrease anxiety or resolve complaints, but may reduce further primary care visits [15].

## Market needs

The total global autoimmune diagnostic tests’ market is predicted to reach $14.2 billion by 2020 [16]. While market growth is due in part to the increased use of multiplex testing in which many analytes from single or multiple samples undergo high-throughput semi-automated screening, there remains an underlying need for only single sample testing for one analyte. For example, there are many circumstances in which a patient presents to primary care or emergency medicine physician with nonspecific symptoms in which the utilization of specific tests such as ANA is particularly informative [11, 17].

## Current clinical needs

Current ANA testing is performed exclusively in centralized clinical laboratories. This is a protracted, labor-intensive, and expensive procedure that can slow the diagnostic process and restricts use in a large segment of the population. Also, the requirement for blood drawing, transport to the testing lab, blood processing, test execution, and communication of results is cumbersome, time consuming, error prone and costly, which detract from its diagnostic value. Primary care physicians recognize delays in test result review as a significant problem affecting quality of care and patient safety—“I wish I has seen this test result earlier”, even raising malpractice concerns [18].

## Point-of-care (POC) ANA testing

POC diagnostics is gaining momentum in different areas of patient care, as its short turnaround time and minimal manual requirements enable quick clinical management decisions [19]. Biosensors based on electrochemical technology offer promise to streamline diagnostic laboratory testing, thereby improving productivity by minimizing costs, time and errors. Electrochemical sensors are particularly well suited for clinically relevant analytes due to their high sensitivity and selectivity with minimal background noise, small size, and power capable by low-voltage battery. Electrochemical POC ANA measurement has potential to meet the expanding testing needs in autoimmune serology, while overcoming the limitations of the ‘gold-standard’ indirect immunofluorescence method (Table 1). In addition to fulfilling the needs in primary care and in urgent/emergency care clinics, POC testing for ANA could be useful in remote or rudimentary settings with the goal of improving diagnostics, enhancing existing test result follow-up protocols, and facilitating timely medical intervention for patients [19].

**Table 1:** Comparison between the standard indirect immunofluorescence method and electrochemical sensor for ANA measurement

Feature	Indirect immunofluorescence	Electrochemical sensor
Cost		
Equipment	Costly fluorescence microscope, infrastructure	Low cost
Individual test	Low–moderate cost	Low cost
Access to providers	Off-site clinical laboratory	Point-of care
Operator expertise	Substantial training needed	Simple to operate
Readout and interpretation	Subjective signal intensity and pattern	Objective continuous digital scale output
Assay time	Substantial processing time (~3 h)	Near real-time data (~20 min)
Result report	Semi-quantitative + pattern	Quantitative; no pattern
Methodology logistics		
Steps	Multiple manipulations	Single-step measurement
Sample autonomy	Usually run in sample batches	Single sample per run
Equipment re-use	No restrictions	Requires cleaning
Antigen substrate		
Antigenic complexity	Fairly comprehensive	More limited
Potential for improvement	None	Readily enhanced at additional cost
Clinical false positives due to DFS70	Present	Absent
Control for non-specific binding	None	Blank substrate

## A prototype biosensor for ANA

We have described development and application of an electrochemical biosensor for rapid quantitation of total ANA having performance characteristics well correlated with ANA titer determination by indirect immunofluorescence in a clinical laboratory [20]. The immunoreactive surface consists of a native autoantigen-rich substrate largely derived from an inexpensive commercially available source that is bound to a porous membrane at high antigen density. By forced transport of the test sample through the membrane loaded with excess autoantigen, antibody–antigen complex formation is complete in less than 3 min. Detection of isotype-specific autoantibodies is achieved by transporting a high-affinity secondary antibody conjugated to peroxidase. Addition of enzyme–substrates results in production of a redox active intermediate transiently captured on the electrode, permitting its detection by amperometry under low voltage. The readout on a digital display is proportional to the amount of antigen-bound antibody. Of particular importance, the autoantigen substrate is devoid of DFS-70/LEDGF, a troublesome antibody-binding ligand of no diagnostic value that detracts from the utility of ANA testing for autoimmune disease [21]. Unlike multiplex testing that is restricted to a limited number of selected autoantigens, the total ANA biosensor employs a complex mix of potential autoantigens and has the plasticity and capacity to accommodate additional antigenic species to produce a universal platform for ANA screening.

## The “Choosing Wisely Initiative” and a biosensor for total ANA

In 2012, the American Board of Internal Medicine Foundation (Philadelphia, PA) launched the “Choosing Wisely” campaign, aiming to advance a dialogue on avoiding wasteful and unnecessary medical tests, treatments, or procedures. Specialty society partners American College of Rheumatology [22] and Canadian Rheumatology Association [23] contributed recommendations for appropriate ANA utilization, stressing that duplicative ANA testing is not warranted if there is high pre-test probability for immune-mediated disease and/or active disease. In this situation, sub-serology testing for antigen-specific autoantibodies can enable stratification of patients into particular autoimmune diseases [22]. However, in situations where there is minimal or even no suggestion of an immune-mediated disease other than vague symptoms, screening for total ANA could be most impactful [11]. Even though the pre-test probability for an ANA-related disease may be low, testing for ANA offers the chance to case-find pre- or early autoimmune disease so that organ damage might be prevented (see ANA ordering pattern #3). To this end, the availability to primary care physicians of a convenient POC ANA testing platform could enhance the goal of improving patient outcomes and reducing health care costs.
